# The effects of curcumin-piperine supplementation on inflammatory, oxidative stress and metabolic indices in patients with ischemic stroke in the rehabilitation phase: a randomized controlled trial

**DOI:** 10.1186/s12937-023-00905-1

**Published:** 2023-12-11

**Authors:** Kosar Boshagh, Fariborz Khorvash, Amirhossein Sahebkar, Muhammed Majeed, Nimah Bahreini, Gholamreza Askari, Mohammad Bagherniya

**Affiliations:** 1 Nutrition and Food Security Research Center, Department of Community Nutrition, School of Nutrition and Food Science, Isfahan University of Medical Sciences, Isfahan, Iran; 2 Neurology Research Center, School of Medicine, Isfahan University of Medical Sciences, Isfahan, Iran; 3 Applied Biomedical Research Center, Mashhad University of Medical Sciences, Mashhad, Iran; 4 Biotechnology Research Center, Pharmaceutical Technology Institute, Mashhad University of Medical Sciences, Mashhad, Iran; 5 Department of Biotechnology, School of Pharmacy, Mashhad University of Medical Sciences, Mashhad, Iran; 6 Sabinsa Corporation, 20 Lake Drive, East Windsor, NJ 08520 USA; 7 Department of Food Science and Technology, School of Nutrition and Food Science, Isfahan University of Medical Sciences, Isfahan, Iran; 8 Anesthesia and Critical Care Research Center, Isfahan University of Medical Sciences, Isfahan, Iran

**Keywords:** Curcumin, Piperine, Ischemic Stroke patients, hs-CRP; lipid Profile, Carotid intima-media thickness

## Abstract

**Background:**

Stroke is a leading cause of death worldwide, which is associated with a heavy economic and social burden. The purpose of this study was to investigate the effects of supplementation with curcumin-piperine combination in patients with ischemic stroke in the rehabilitation stage.

**Methods:**

In this randomized controlled trial, 66 patients with stroke were randomized into two groups receiving curcumin-piperine tablets (500 mg curcumin + 5 mg piperine) and matched placebo tablets for 12 weeks. High-sensitivity C-reactive protein (hs-CRP), carotid intima-media thickness (CIMT), thrombosis, total antioxidant capacity (TAC), lipid profile, anthropometric indices, blood pressure, and quality of life were assessed before and after the intervention. Statistical data analysis was done using SPSS22 software.

**Results:**

A total of 56 patients with a mean age of 59.80 ± 4.25 years completed the trial. Based on ANCOVA test, adjusted for baseline values, curcumin-piperine supplementation for 12 weeks resulted in significant reductions in serum levels of hs-CRP (*p* = 0.026), total cholesterol (TC) (*p* = 0.009), triglycerides (TG) (*p* = 0.001), CIMT (*p* = 0.002), weight (*P* = 0.001), waist circumference (*p* = 0.024), and systolic and diastolic blood pressure (*p* < 0.001), and a significant increase in TAC (*p* < 0.001) in comparison to the placebo. Pain score significantly increased in both groups; however, its increase was significantly higher in the placebo group compared with the intervention group (*p* = 0.007). No significant changes were observed between the two groups in terms of serum fibrinogen, low-density lipoprotein (LDL), high-density lipoprotein (HDL), and quality of life indices.

**Conclusion:**

Curcumin-piperine supplementation had beneficial effects on CIMT, serum hs-CRP, TC, TG, TAC, and systolic and diastolic blood pressure in patients with ischemic stroke in the rehabilitation stage.

## Introduction

Stroke is a leading cause of death worldwide [1, 2], and a major reason for physical and mental disabilities in societies [3]. Approximately 16 million primary strokes occur annually in the world, resulting in 5.7 million deaths. Although stroke is a global epidemic, about 85% of all deaths from strokes occur in low- or middle-income countries [4]. Diabetes, hypertension, smoking, hyperlipidemia, heart disease, obesity, and physical inactivity are modifiable risk factors, while old age, male gender, family history, and race are considered non-modifiable risk factors of stroke [5, 6].

Following a stroke, a cascade of molecular events including increased extracellular glutamate concentration, increased intracellular calcium levels, and oxidative stress are initiated [3]. Oxidative stress is caused by the overproduction of reactive oxygen species (ROS) and reactive nitrogen species (RNS), which can lead to detrimental effects such as inhibiting mitochondrial function, increasing calcium levels, reperfusion damage, and inflammation [7]. Inflammation of neurons destroys the blood-brain barrier, which can culminate in the formation of brain edema, and finally cell death [8]. As a result, patients show different degrees of neurological complaints such as cognitive disorders, learning and memory deficits, inflammatory responses, motor, sensory, vision, and balance disorders, and functional defects in various organs, affecting regular daily activities [9].

Stroke causes changes in the patients’ lifestyle and ultimately affects their quality of life [10]. Quality of life is a broad concept that includes physical, psychological, social, and personal beliefs [11]. Studies conducted on patients with stroke have shown poor quality of life in these patients [12]. In recent years, measuring the quality of life and improving it has been one of the primary goals of treatment. The patient’s response to the disease can be evaluated by measuring the quality of life score [13]. Currently, drugs such as glucocorticoids are frequently used to reduce inflammation in patients with stroke, while these drugs have several side effects that have led researchers to look for newer effective therapies with fewer side effects [14]. Curcumin is one of the main components of the rhizome of *Curcuma longa* L. plant. This polyphenolic compound makes up about 2 to 8% of turmeric compounds [15, 16]. Curcumin has been shown to serve as a promising factor for preventing stroke and improving its complications in animal studies [17].

Curcumin has many pharmacological activities that are relevant to the treatment of stroke [18, 19]. Preclinical studies have shown the protective effects of curcumin against stroke due to the anti-inflammatory and antioxidant, and anti-ischemic properties of the molecule [20, 21].

Studies showed that apoptosis during ischemia/reperfusion plays an important role in stroke-related brain damage [22]. Curcumin exerts its neuroprotective effect by regulating cell apoptosis and increasing neurogenesis [17]. This phytochemical downregulates Bax (Bcl-2 associated X-protein) and caspase-3, and prevents the decrease in Bcl-2 (B-cell lymphoma-2) level, thereby protecting against neuronal apoptosis and Bax activation during cerebral ischemia [23]. Animal studies have shown that post-stroke administration of curcumin can significantly reduce lipid peroxidation, mitochondrial dysfunction, glial activation, and infarct volume. In addition, curcumin improves cognitive deficits and motor activity [24].

Abdominal obesity, hyperlipidemia, hypertension, inflammation, and oxidative stress are considered risk factors for ischemic stroke; treatment of them is mandatory for both prevention of stroke and reducing the risk of mortality in patients with ischemic stroke [25–30]. Although the efficacy of curcumin in reducing stroke indices among patients with stroke has not been assessed to date, a recent systematic review and meta-analysis showed that curcumin had promising effects on these risk factors [31–36]. Thus, based on this evidence, supplementation with curcumin in patients with ischemic stroke might have beneficial effects, which may lead to improvement of their overall health and reduction of mortality.

Curcumin, as a strong anti-inflammatory agent, inhibits the activation of NF-KB and ensuing expression of various inflammatory cytokines, including TNF-α, IL-8, IL-6, and IL- 1, and reduces the expression of CRP and inflammatory enzymes such as cyclooxygenase 2 (COX-2) and inducible nitric oxide synthase (iNOS) [37, 38]. Curcumin exhibits its antioxidant effects by inhibiting superoxide radicals, hydrogen peroxide, and nitric oxide, and increasing the expression of antioxidant proteins through Nrf2 [38–40]. Moreover, it increases the activity of many antioxidant enzymes such as catalase, superoxide dismutase, heme oxygenase, and glutathione peroxidase, thus preventing lipid peroxidation [39].

Curcumin reduces triglycerides by decreasing the activity of lipoprotein lipase [41]. The possible mechanism of curcumin in improving dyslipidemia is increasing cholesterol catabolism by increasing the activity of the liver cholesterol 7-hydroxylase enzyme, which in turn inhibits cholesterol synthesis by inhibiting the HMG-COA reductase enzyme [42]. The effect of curcumin on lowering blood pressure is applied through the effect on vascular function and antioxidant activity of this phytochemical, which includes inhibiting reactive oxygen species (ROS), increasing the bioavailability of nitric oxide, and improving the glutathione defense system [43].

Curcumin regulates the Janus Kinase enzyme (JNK), which has been shown to play an essential role in the pathogenesis of obesity [44, 45]. Curcumin may also inhibit the 11β-HSD1 enzyme that activates cortisol [46]. Higher cortisol concentrations in adipocytes cause central obesity [47]. Another possible mechanism of curcumin’s effect on obesity is related to its effect on hormones. Previous meta-analyses reported that curcumin consumption can decrease leptin and increase adiponectin levels, thereby modulating appetite and energy homeostasis [48].

Despite its high therapeutic potential, curcumin is limited for medical purposes due to its low aqueous solubility, low bioavailability, and rapid degradation [49]. To improve its pharmacokinetic features, co-administration of curcumin with piperine has been introduced as an alternative. Piperine, which is a naturally occurring alkaloid from pepper, has been shown to increase the bioavailability of curcumin and reduce its glucuronidation [38].

It seems that curcumin has several unique properties to improve the health condition of patients with ischemic stroke. However, to the best of our knowledge, there has been no clinical trial investigating the efficacy of curcumin on these patients to date. Thus, the purpose of this study was to investigate the effects of supplementation with curcumin-piperine combination in patients with ischemic stroke in the rehabilitation stage.

## Methods

### Study design and participants

This study was a parallel, double-blind, placebo-controlled clinical trial to evaluate the effectiveness of curcumin-piperine supplementation in patients with stroke. Participants were recruited from Imam Musa Sadr Clinic, affiliated with Isfahan University of Medical Sciences, Isfahan, Iran from December 2020 to December 2021. The Ethics Committee of Isfahan University of Medical Sciences approved this project with the approval number IR.MUI.RESEARCH.REC.1399.593. This trial was registered with the Iranian Registry of Clinical Trials with ID: IRCT20121216011763N48. This study was performed based on the Helsinki Declaration. Written informed consent was obtained from all participants before the study.

### Participants

Patients with Ischemic stroke aged 20–65 years, who were in the rehabilitation phase 3–6 months following the active stroke attack were included in this study based on the following criteria: diagnosis of thrombotic and embolic stroke through a computerized tomography (CT) scan and Magnetic resonance imaging (MRI), having body mass index (BMI) between 18.5 and 35, the NIH Stroke Scale/Score (NIHSS) 5–24 [50, 51], non-pregnancy and lactation, no smoking and alcohol use, not following a special diet or exercise program in the past three months, not taking warfarin, antioxidants, multivitamins, herbal supplement, and omega-3 supplements, and not suffering from malignant diseases, cancers, fatty liver, diabetes and endocrine disorders, heart, lung and kidney diseases, cirrhosis, hepatitis, and immune system disorders. Exclusion criteria included any history of allergy to herbal products such as turmeric and pepper, recurrent stroke, unwillingness to continue the study treatment, observing any side effects, and presence of autoimmune infectious and any other chronic disease conditions.

### Trial randomization and blinding

Eligible patients were randomly divided and matched on age and gender in a ratio of 1:1 into two groups receiving curcumin piperine or placebo. To create a randomization list, we used the following website “https://www.sealedenvelope.com/simple-randomiser/v1/lists”. Assignment sequences were conducted by an independent statistician applying a table of random numbers. Then, until the end of the evaluation of eligibility criteria, these were kept in opaque, sealed, numbered envelopes. Treatment assignments were hidden from patients and researchers until the end of data analyses. Curcumin piperine and its placebo tablets were produced by Sami Labs Limited (Bangalore, India). All tablets were packed by the company in similar boxes (codes A and B). The appearance properties of the tablets were completely similar, the tablets had the same color, size, and odor. Patients, physicians, researchers, laboratory staff, and other staff associated with the study were blinded until the end of the trial and the complementation of analyses.

### Intervention

A trained nutritionist (K.B.) under the supervision of an expert neurologist (F.KH.) implemented the study. Sixty-six patients with stroke were divided into two groups: (i) the intervention group (n = 33) received a tablet containing 505 mg/day curcumin-piperine with 95% purity, (each tablet provided 500 mg curcumin and 5 mg piperine purely) for 12 weeks, and (ii) patients in the control group received a placebo tablet matched with the intervention in terms of size, shape, and color (n = 33) for the same duration. Patients were asked to take tablets one hour after eating breakfast. Any remaining supplements were counted at the end of the intervention. If more than 10% of the supplement was not taken by participants during the study, they were excluded from the study. Compliance was determined from the capsule count in the bottles at the end of the trial, and patients should at least consume 90% of tablets within the study [52]. The patients were reminded about the regular consumption of supplements through phone calls and SMS messages. All patients received their routine and usual treatment approach under the supervision of their neurologist.

Intervention with 505 mg curcumin-piperine for 12 weeks was used because this study was the first to assess the efficacy of curcumin-piperine in patients with stroke, and the same dose has been the most frequently used dose with documented efficacy and safety in previous trials. Although curcumin is safe at higher doses [19, 53], we chose the lowest effective dose based on previous clinical trials. In addition, in a previous study, it was shown that the dose of 505 mg curcumin-piperine for 12 weeks had beneficial effects on some clinical and biochemical indices of individuals in different populations [54].

### Sample size calculation

According to a previous article [55], by placing alpha error values of 5% (Z = 1.96), beta error of 20%, Power = 80% (Z = 0.84), and Δ = 0.7, the sample number for each group was calculated as 33 participants per group.

### Assessment of variables

#### Demographic and anthropometric parameter assessment

Demographic variables including sex, age, education, medical history, marital status, and family history of stroke were collected from all participants by completing a general information questionnaire. Weight was measured with 0.1 kg accuracy, and height was measured with 0.1 cm accuracy. Waist circumference was measured in the standing position (at the shortest distance between the last rib and the iliac spine). Then, body mass index (BMI) was calculated by dividing weight (kg) by the square of height (cm).

#### Assessment of clinical measures

Blood samples (10 ml) were collected after 10–12 h of fasting and this process was repeated after the end of the intervention. Triglycerides (TG), serum cholesterol, high-density lipoprotein (HDL), and low-density lipoprotein (LDL) were determined using an enzyme method. Total antioxidant capacity (TAC) was measured using a commercial kit (Kiazist, Hamedan, Iran). Fibrinogen levels and high sensitivity C-reactive protein (hs-CRP) were measured using standard ELISA kits (AUDIT kit, Tehran, Iran), in the Laboratory of Biochemistry, Chamran Hospital, Isfahan, Iran. Blood pressure was measured using a mercury sphygmomanometer after the patient had been sitting for 10 min. Before taking blood pressure, patients were asked about smoking or drinking coffee in the previous 2 h. The carotid intima-media thickness (CIMT) vascular index was measured by Doppler ultrasound [56], and by a single expert neurologist for all patients (F.KH.).

#### Dietary intake and physical activity assessment

To evaluate the nutritional status of the patients, food records (2-week days and 1-weekend) were used at the beginning of the study, 6 (middle) and 12 (end) weeks of the intervention, and the average of three periods were calculated and reported [57, 58]. Using the information obtained from these records, energy, and macronutrient intake were calculated by Nutritionist IV software. Considering the age range of the subjects and the special conditions of these patients who are mostly unable to do physical activity, it is not necessary to use the physical activity register, and the physical activity of the participants was measured through a general information questionnaire.

#### Quality of life assessment

The SF-36(short-form 36 questionnaire) quality of life questionnaire was completed by the participants before and after the intervention. The SF-36 quality of life questionnaire is a tool that is widely used and has been validated to measure the quality of life of patients with stroke. This questionnaire is a self-expressive tool and includes 36 items in 8 scales including physical functioning, role functioning/physical, role functioning/emotional, energy/fatigue, emotional well-being, pain, social functioning, and general health. These 8 scales include both physical and mental components. The score of each subscale is from 0 to 100 and a higher score closer to 100 indicates a better quality of life [59]. The validity and reliability of the SF-36 questionnaire have been conducted in Iran, and the Persian version of this questionnaire has good validity and reliability [60].

### Statistical analysis

The Skewness index and q-q-plot were used to evaluate the normality of variables. The intergroup comparison was done with paired t-test and the between-group comparison was done with an independent t-test. Between-group comparisons for qualitative traits were performed based on the chi-square test. Between-group comparisons were performed to control for confounding factors through analysis of covariance (ANCOVA). Data were reported as mean ± standard deviation (SD) or frequency (percentage). Statistical data analysis was done using SPSS22 software. A *p*-value less than 0.05 (two-sided) is considered a statistically significant.

## Results

**Fig. 1:**
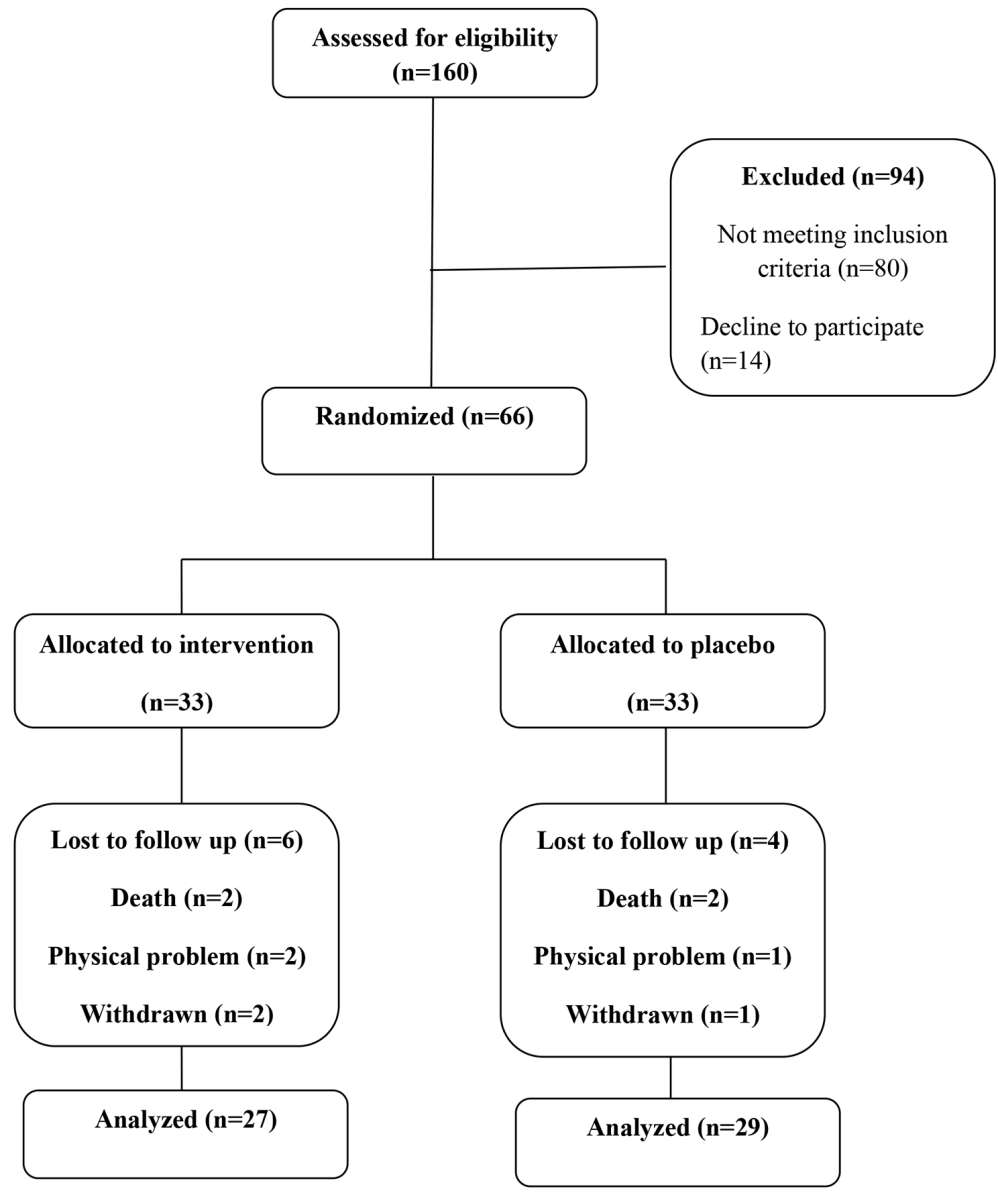
Patient’s flow diagram

Among the 160 patients with stroke, 66 met the inclusion criteria and were included in this clinical trial. Six patients were excluded from the curcumin-piperine group (2 due to death, 2 due to physical problems (inability to move), and 2 due to unwillingness to continue the study), and 4 patients were excluded from the placebo group (2 due to death, 1 due to physical problems (inability to move) and 1 due to unwillingness to continue the treatment). Finally, 56 patients (27 subjects in the curcumin-piperine group and 29 persons in the control group) completed the trial (Fig. 1).

No adverse effects were reported throughout the intervention and the intervention was safe. The curcumin-piperine group had an average age of 59.48 ± 5.15 and the control group was 60.12 ± 3.12 years. As shown in Table 1, there were no significant differences between the two groups in terms of socio-demographic variables. In addition, no significant differences were observed in macronutrients and micronutrient consumption throughout the trial between the two groups except for vitamin E (*p* = 0.001) **(**Table 2**)**.

**Table 1:** Baseline characteristics of study subjects

Variable			Curcumin-piperine group	Placebo group	*P* ^*1*^
Age (year)			59.48 ± 5.15	60.12 ± 3.12	0.548
Weight(kg)			68.86 ± 13.93	73.09 ± 12.74	0.203
Height(cm)			160.66 ± 6.59	163.96 ± 8.21	0.076
Body mass index(kg/m^2^)			26.61 ± 4.84	27.04 ± 3.79	0.689
Waist circumference(cm)			89.12 ± 12.57	91.57 ± 11.11	0.404
Family history of stroke, *n* (%)			18 (54.54%)	16 (48.48%)	0.622
History of disease (diabetes, hypertension and …), *n* (%)			32 (96.96%)	31 (93.93%)	0.555
Gender	Male, *n* (%)		11 (33.33%)	15 (45.45%)	0.314
	Female, *n* (%)		22 (66.66%)	18 (54.54%)	
Marital status	Married, *n* (%)		31 (93.93%)	32 (96.96%)	0.602
	Single, *n* (%)		1 (3.03%)	0	
	Widow, *n* (%)		1 (3.03%)	1 (3.03%)	
Education	Under diploma *n* (%)		22 (66.66%) 11 (33.33%)	21 (63.63%) 12 (36.36%)	0.796
	Diploma or higher *n* (%)				
Physical activity		Yes, *n* (%)	1 (3.03%)	5 (15.15%)	0.087
		No, *n* (%)	32 (96.96%)	28 (84.84%)	

*P*^1^ values obtained from independent T tests for continuous and Chi-square for categorical variables between the two groups

**Table 2:** Dietary intake of the participants throughout the study

Variable	Curcumin-piperine group	Placebo group	*P* ^*1*^
Energy (kcal/day)	1427.5287 ± 191.81503	1500.6511 ± 239.63143	0.215
Protein (g/day)	69.1792 ± 11.05807	69.3767± 16.83638	0.959
Carbohydrate (g/day)	196.5598 ± 36.11480	212.6983 ± 47.29984	0.159
Fat (g/day)	45.4769 ± 8.68696	49.6709 ± 8.49561	0.073
Saturated fat(g)	15.3685 ± 3.10594	15.4402 ± 2.77602	0.928
Fiber (g/day)	27.3572 ± 10.68200	28.8329 ± 8.64451	0.571
Potassium (mg)	3128.7037 ± 1087.15465	3316.5944 ± 867.62391	0.476
Vit C (mg)	99.9607 ± 55.31416	134.1394 ± 176.93561	0.341
Calcium (mg)	1053.3666 ± 417.95519	1109.3948 ± 344.18040	0.585
Fe (mg)	30.0434 ± 24.45524	33.2810 ± 20.60255	0.593
Vit E (mg)	8.2343 ± 2.04844	11.2707 ± 4.07786	0.001
Zinc (mg)	10.3705 ± 2.37966	10.7488 ± 2.11432	0.531
Selenium (µg)	95.3898 ± 23.70947	107.9005 ± 74.03888	0.405

*P*^1^ Obtained from independent t test

As shown in Table 3, based on the paired T-test (within the group), there was a significant decrease in hs-CRP (*p* < 0.001), fibrinogen (*p* < 0.001), TG (*p* = 0.017), TC (*p* = 0.034), CIMT (*p* < 0.001), systolic and diastolic blood pressure (*p* < 0.001) and a significant increase in TAC (*p* < 0.001), HDL (*p* < 0.001) in the curcumin-piperine group compared with baseline. However, in the placebo group, compared with baseline, weight (*p* = 0.013), LDL (*p* = 0.024), HDL (*p* < 0.001), and TC (*p* = 0.003) significantly increased. On the other hand, systolic (*p* = 0.019) and diastolic (*p* = 0.026) blood pressure significantly decreased in the placebo group at post-intervention compared with baseline.

**Table 3:** Main study indicators before and after the intervention in the curcumin-piperine and placebo groups

Variable	Curcumin-piperine group				placebo group				*P* ^2^	*P* ^3^
	before	after	changes	*P* ^1^	before	after	changes	*P* ^1^		
CIMT (mm)	0.94 ± 0.13	0.85 ± 0.13	-0.09 ± 0.11	< 0.001	1.00 ± 0.13	0.98 ± 0.13	-0.02 ± 0.06	0.061	0.002	0.001
hs-CRP (mg/L)	2.48 ± 5.82	0.48 ± 0.74	-2.00 ± 5.72	< 0.001	2.18 ± 4.95	2.39 ± 5.11	0.20 ± 3.85	0.772	0.026	0.015
LDL (mg/dL)	111.15 ± 41.84	108.40 ± 40.45	-2.74 ± 31.73	0.657	94.03 ± 30.49	113.27 ± 40.45	19.24 ± 43.46	0.024	0.139	0.270
HDL (mg/dL)	46.33 ± 13.76	73.07 ± 22.61	26.74 ± 17.85	< 0.001	49.44 ± 17.66	68.79 ± 18.46	19.34 ± 20.15	< 0.001	0.212	0.439
TG (mg/dL)	174.55 ± 99.98	134.18 ± 62.42	40.37 ± 82.45	0.017	169.0 ± 84.36	179.24 ± 72.94	10.17 ± 48.46	0.268	0.001	0.022
TC (mg/dL)	186.40 ± 61.37	162.07 ± 36.48	24.33 ± 56.36	0.034	155.7 ± 34.78	178.58 ± 44.87	22.79 ± 37.74	0.003	0.009	0.020
Fibrinogen (mg/dL)	474.11 ± 104.46	402.85 ± 60.23	71.25 ± 88.37	< 0.001	350.24 ± 113.10	376.13 ± 107.79	25.8 ± 101.4	0.180	0.223	0.313
SBP (mmHg)	13.40 ± 2.00	12.11 ± 0.80	-1.29 ± 1.65	< 0.001	13.31 ± 1.51	12.72 ± 0.64	-0.58 ± 1.26	0.019	< 0.001	< 0.001
DBP (mmHg)	9.29 ± 1.63	8.33 ± 0.67	-0.96 ± 1.09	< 0.001	9.31 ± 1.19	8.89 ± 0.77	-0.41 ± 0.94	0.026	< 0.001	< 0.001
TAC (mmol/ml)	2.81 ± 0.19	2.88 ± 0.19	0.07 ± 0.06	< 0.001	2.85 ± 0.19	2.84 ± 0.19	-0.01 ± 0.01	0.404	< 0.001	< 0.001
Weight (kg)	69.12 ± 14.64	68.96 ± 14.27	-0.16 ± 0.59	0.167	73.82 ± 13.26	74.14 ± 13.16	0.31 ± 0.64	0.013	0.001	0.015
WC (cm)	89.92 ± 13.13	89.80 ± 12.97	-0.12 ± 0.32	0.059	92.75 ± 11.11	92.81 ± 11.20	0.05 ± 0.26	0.310	0.024	0.086
BMI (kg/m^2^)	26.88 ± 5.04	26.82 ± 4.89	-0.05 ± 0.23	0.209	27.33 ± 3.77	27.10 ± 4.25	-0.22 ± 1.85	0.524	0.665	0.539

Variables are expressed as mean ± SD Abbreviations: CIMT, carotid intima media thickness; hs-CRP, high sensitive C reactive protein; LDL, low-density lipoprotein; HDL, high-density lipoprotein; TG, Triglyceride; TC, serum cholesterol; SBP, systolic blood pressure; DBP, diastolic blood pressure; TAC, total antioxidant capacity; WC, waist circumference; BMI, body mass index

*P*^1^ Obtained from paired t test. *P*^2^ Obtained from ANCOVA adjusted for baseline values. P^3^ Obtained from ANCOVA adjusted for baseline values, and vitamin E intake

In comparison to placebo (between group analysis), supplementation with curcumin piperine for 12 weeks resulted in a significant reduction in CIMT (*p* = 0.002), serum hs-CRP (*p* = 0.026), TC (*p* = 0.009), TG (*p* = 0.001), weight (*p* = 0.001), waist circumference (*p* = 0.024) and systolic and diastolic blood pressure (*p* < 0.001), and a significant increase in TAC level (*p* < 0.001) (Table 3). Since intake of vitamin E during the study was significantly higher in the control group compared with the intervention group, we conducted further analysis, and in addition to the baseline values, the intake of vitamin E was adjusted in the ANCOVA test. As shown in Table 3, however, this analysis did not change the findings except for waist circumference, which difference between groups was not statistically significant, adjusting for both baseline value and vitamin E intake (*p* = 0.086).

In assessing the quality of life indicators, physical functioning, role functioning/ physical energy/fatigue, emotional well-being, and social functioning significantly increased in both intervention and control groups (Table 4). However, there were no significant intergroup differences for all of these variables. Nevertheless, there were no significant changes in the variables of general health and role functioning/emotion in both the intervention and control groups. Finally, the pain variable was significantly increased in the curcumin-piperine group (*p* = 0.005) and placebo group (*p* < 0.001). Comparison between groups showed that the increase in pain was higher in the placebo group in comparison to the curcumin-piperine group (*p* = 0.007). Since the intake of vitamin E during the study was significantly higher in the control compared with the intervention group, we conducted further analysis, and in addition to the baseline values, the intake of vitamin E was adjusted in the ANCOVA test. As shown in Table 4, this analysis did not change the findings.

**Table 4:** Quality of Life Indicators before and after the intervention in the curcumin-piperine and placebo groups

Variable	Curcumin-piperine group				placebo group				*P* ^2^	*P* ^3^
	Before	After	Changes	*P* ^1^	Before	After	Changes	*P* ^1^		
Physical Functioning	65.37 ± 12.85	66.29 ± 12.52	0.92 ± 2.41	0.057	67.24 ± 9.31	68.96 ± 8.59	1.72 ± 2.41	0.001	0.145	0.176
Role functioning/ physical	81.48 ± 17.80	88.88 ± 14.43	7.40 ± 11.63	0.003	82.75 ± 16.50	88.27 ± 15.07	5.51 ± 10.29	0.007	0.565	0.596
Role functioning/ emotional	92.59 ± 14.12	95.06 ± 12.06	2.46 ± 8.89	0.161	94.25 ± 15.60	94.25 ± 15.60	0	0.99	0.160	0.216
Energy/ fatigut	61.11 ± 7.25	63.14 ± 6.81	2.03 ± 2.50	< 0.001	61.40 ± 8.13	63.01 ± 6.92	1.60 ± 2.33	0.001	0.503	0.438
Emotional well being	63.77 ± 7.28	64.96 ± 6.95	1.18 ± 1.86	0.003	62.40 ± 7.36	63.50 ± 7.67	1.10 ± 1.81	0.003	0.806	0.462
Social functioning	85.18 ± 16.99	89.81 ± 14.72	4.62 ± 7.86	0.005	87.50 ± 11.57	90.94 ± 8.77	3.44 ± 7.39	0.018	0.771	0.612
Pain	89.45 ± 15.49	92.87 ± 14.17	3.41 ± 5.85	0.005	90.77 ± 11.49	97.75 ± 5.60	6.98 ± 7.51	0 < 001	0.007	0.006
General health	62.03 ± 9.32	62.22 ± 9.64	0.18 ± 1.68	0.574	59.82 ± 7.96	60.34 ± 8.01	0.51 ± 1.54	0.083	0.741	0.441

Values are presented as mean ± SD. *P*^1^ Obtained from paired t test. *P*^2^ Obtained from ANCOVA adjusted for baseline values. *P*^3^ Obtained from ANCOVA adjusted for baseline values, and vitamin E intake

## Discussion

The results of this study showed that 12 weeks of curcumin-piperine supplementation reduced CIMT, hs-CRP, TC, TG, systolic and diastolic blood pressure, weight, waist circumference, and increased TAC in patients with stroke. Based on our knowledge, this study is the first clinical trial that examined the effect of curcumin-piperine on clinical as well as biochemical risk factors in patients with stroke. The findings of this study might help improve the health status of stroke patients.

We observed a significant reduction in CIMT in patients with stroke supplemented with curcumin-piperine for 12 weeks. The authors were unable to find any previous trial that investigated the effects of curcumin supplementation on CIMT. However, in one study, the relationship between oxidative stress indices and antioxidant levels with CIMT in patients was explored in patients undergoing hemodialysis. In the mentioned study, a positive correlation was found between CIMT and thiobarbituric acid reactive substances (TBARS) and nitrate-to-nitrite ratio as markers of oxidative stress, while there was a negative correlation between CIMT and superoxide dismutase and catalase as antioxidants, suggesting the beneficial effects of antioxidants on the CIMT [61]. Since curcumin is a compound with strong antioxidant properties [62], its intake seems to be effective in reducing CIMT.

Curcumin exerts its antioxidant effects by inhibiting superoxide radicals, hydrogen peroxide, and nitric oxide. Curcumin increases the activity of many antioxidant enzymes such as catalase, heme oxygenase, superoxide dismutase, and glutathione peroxidase, thereby preventing lipid peroxidation [39]. In the present study, the consumption of curcumin-piperine increased TAC, which is consistent with the results of previous studies but inconsistent with the results reported by Salehi et al. [63] and Bakhtiari et al. [64]. The reasons for this discrepancy might be different sample groups and different preparations of curcumin compared with the present study.

This study showed a reduction in serum hs-CRP levels after 12 weeks of curcumin-piperine supplementation. The results of Gorabi et al.‘s [65] study were consistent with the results of our study. In the meta-analysis by Gorabi et al., nine studies examined the effect of curcumin on CRP (C-reactive protein) and 23 studies on hs-CRP in patients with inflammatory conditions. CRP significantly decreased after > 10 weeks of intervention compared with the control group, and hs-CRP also significantly decreased compared with the control group.

It has been shown that curcumin reduces inflammatory responses by reducing the activities of cyclooxygenase-2, lipoxygenase, and nitric oxide synthase enzymes, and the production of inflammatory cytokines including tumor necrosis factor-alpha (TNF-α) as well as interleukins 1, 2, 6, 8, and 12. Curcumin also inhibits nuclear factor κB (NF-κB), and NLRP3 inflammasome as key inducers of inflammation [66, 67].

The present study showed a decrease in systolic and diastolic blood pressure after 12 weeks of curcumin-piperine supplementation in comparison to placebo, which is in line with the results of Khajehdehi et al. [68] and Jazayeri et al. [69]. In Jazayeri et al.‘s study on 84 overweight/obese patients with non-alcoholic fatty liver disease (NAFLD), daily intake of 80 mg of nano-curcumin for 3 months significantly reduced systolic blood pressure. Moreover, in the study of Khajehdehi et al. on 24 patients with recurrent or resistant lupus nephritis, 500 mg of curcumin for 3 months was administered and the results showed a significant reduction in systolic blood pressure. In the meta-analysis study by Hadi et al. [32], a total of 11 studies and 732 eligible participants were included. The results showed that curcumin/turmeric supplementation for ≥ 12 weeks led to a significant reduction in systolic blood pressure, without any impact on diastolic blood pressure.

It is suggested that the effect of curcumin on lowering blood pressure is applied through the effect on antioxidant activity and vascular function of this phytochemical, which includes inhibiting reactive oxygen species (ROS), increasing the nitric oxide bioavailability, and improving the glutathione defense system [43]. It has also been proposed that curcumin reduces blood pressure by inhibiting the level of ACE(Acetylcholine) and angiotensin-2, and protects the cholinergic system of the brain by inhibiting the activity of AChE (Acetylcholinesterase) [70].

We found that curcumin-piperine intake can lower serum TC and TG concentrations. The probable mechanism of curcumin in improving dyslipidemia is reducing the activity of lipoprotein lipase [41], suppressing the activity of fatty acid synthase (FAS), increasing the activity of fatty acid beta-oxidation [71], and increasing cholesterol catabolism through increasing the activity of the liver enzyme cholesterol 7-hydroxylase, which in turn, inhibits cholesterol synthesis by inhibiting the enzyme HMG-COA reductase [72].

Results of a previous meta-analysis in which 26 clinical trials comprising 1890 participants were included were in line with our study [73], as it showed that curcumin supplementation significantly reduced TG and TC levels, but had no significant effect on LDL and HDL levels.However, in an earlier meta-analysis by Sahebkar et al. [74] including five clinical trials with 223 participants, curcumin supplementation had no significant effect on lipid profile indices.

Other results from this study showed improvements in fibrinogen after 12 weeks of curcumin-piperine supplementation, although these changes were not significant compared with the placebo group. Fibrinogen is involved in platelet aggregation, endothelial cell damage, and plasma viscosity. These mechanisms play a central role in thrombosis, and thrombosis is the main cause of ischemia [75]. Curcumin has multiple positive in modulating platelet aggregation, coagulation, and fibrinolysis [76, 77] and appears to be effective in reversing high levels of blood fibrinogen. Thus far, only limited studies have investigated the effects of curcumin supplementation on fibrinogen levels in patients with stroke or other conditions. In a clinical report by Ramirez Bosca et al., it was shown that daily consumption of 20 mg of curcumin for 15 days in 8 people with high levels of fibrinogen resulted in a significant reduction in the level of fibrinogen [78]. The insignificant result observed in our study could be related to the small sample size.

Other results in this study showed that curcumin-piperine supplementation decreased weight and waist circumference compared with the control group, but BMI did not decrease significantly compared with the control group. In a meta-analysis by Akbari et al., a total of 21 clinical trials including 1604 people were included and the results showed that curcumin significantly reduced weight, BMI, and waist circumference [79]. However, the meta-analysis by Jafarirad et al., including 8 trials with 449 participants, did not show any significant effect of curcumin on weight, BMI, and waist circumference [48].It has been revealed that the Janus Kinase (JNK) enzyme is regulated by curcumin, which plays an essential role in obesity pathogenesis. In addition, the 11βHSD1 enzyme that activates cortisol might be inhibited by curcumin. Central obesity can be developed by a higher concentration of cortisol in fat cells. Moreover, curcumin can reduce obesity through several mechanisms such as inhibition of adipogenesis in the early stages through suppression of the peroxisome proliferator-activated receptor c (PPAR-c) and increase of the activation of protein kinase through monophosphate following lipolysis [80].

In the present study, curcumin-piperine supplementation was associated with a significant improvement in physical functioning, role functioning/physical, energy/fatigue, emotional well-being, and social functioning, although these changes were not significant compared with the placebo group. Moreover, curcumin caused less increase in pain compared with the control group. Curcumin’s analgesic properties have been previously reported in a meta-analysis of randomized controlled trials [81].These analgesic effects can be attributed to the anti-inflammatory effects of this phytochemical mediated *via* inhibiting pro-inflammatory mediators such as lipoxygenase, nitric oxide, and interleukin-6 [18].

This study is the first randomized, double-blind, placebo-controlled clinical trial investigating the effect of curcumin in stroke patients. Limitations of this study need to be considered. Most of the patients were > 65 years of age and the study was conducted concurrently with the Covid-19 pandemic, which made it difficult to sample and reach the appropriate sample size. In addition, we have no long-term follow-up up and due to the ethical issue, evaluation of the efficacy of curcumin-piperine alone is impossible.

## Conclusion

Results of the current study indicated that curcumin-piperine co-supplementation has beneficial effects on CIMT, systolic and diastolic blood pressure as well as serum levels of hs-CRP, TC, TG, and TAC. No significant changes were observed between the two groups in terms of serum fibrinogen, LDL, HDL, and most of the quality-of-life indicators. More well-designed and long-term randomized controlled trials are needed to confirm the present results.

## Data Availability

The data that support the findings of this study are available from the corresponding author (MB) upon reasonable request.
